# Rho-Kinase/ROCK as a Potential Drug Target for Vitreoretinal Diseases

**DOI:** 10.1155/2017/8543592

**Published:** 2017-05-10

**Authors:** Muneo Yamaguchi, Shintaro Nakao, Mitsuru Arima, Iori Wada, Yoshihiro Kaizu, Feng Hao, Shigeo Yoshida, Koh-hei Sonoda

**Affiliations:** Department of Ophthalmology, Graduate School of Medical Sciences, Kyushu University, 3-1-1 Maidashi, Higashi-Ku, Fukuoka 812-8582, Japan

## Abstract

Rho-associated kinase (Rho-kinase/ROCK) was originally identified as an effector protein of the G protein Rho. Its involvement in various diseases, particularly cancer and cardiovascular disease, has been elucidated, and ROCK inhibitors have already been applied clinically for cerebral vasospasm and glaucoma. Vitreoretinal diseases including diabetic retinopathy, age-related macular degeneration, and proliferative vitreoretinoapthy are still a major cause of blindness. While anti-VEGF therapy has recently been widely used for vitreoretinal disorders due to its efficacy, attention has been drawn to new unmet needs. The importance of ROCK in pathological vitreoretinal conditions has also been elucidated and is attracting attention as a potential therapeutic target. ROCK is involved in angiogenesis and hyperpermeability and also in the pathogenesis of various pathologies such as inflammation and fibrosis. It has been expected that ROCK inhibitors will become new molecular target drugs for vitreoretinal diseases. This review summarizes the recent progress on the mechanisms of action of ROCK and their applications in disease treatment.

## 1. Introduction

**Figure 1:**
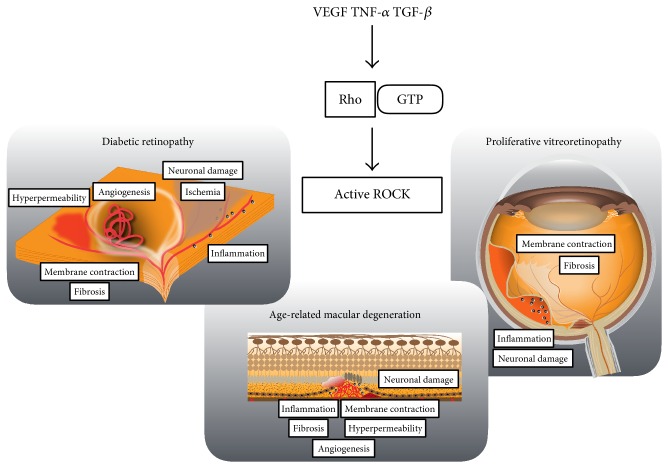
ROCK-activated vitreoretinal diseases. ROCK activation is involved in the pathology of retinal vitreous diseases such as diabetic retinopathy, age-related macular degeneration, and PVR, termed as “ROCK-activated diseases.” In each disease, the Rho-ROCK signaling pathway is activated by various cytokines, implicating ROCK in various pathology. Therefore, ROCK is a potential therapeutic target for these vitreoretinal diseases.

Rho-associated kinase (Rho kinase/ROCK), identified as a Rho GTP-binding protein, is a downstream effector of the small GTP-binding protein Rho [1–5]. Two isoforms, ROCK1 (also known as ROK*β* or p160ROCK) and ROCK2 (known as ROK*α*), were isolated as Rho-A-GTP interacting proteins [6]. The Rho/ROCK signaling pathway is implicated in various cellular functions, such as cell proliferation, migration, and contraction [7], and has been reported to be crucial for cardiovascular diseases, central nervous disorders, and cancer. Therefore, ROCK has attracted attention as a therapeutic target for various diseases [8, 9]. Recent studies have implicated Rho/ROCK signaling in both physiological and pathological ophthalmology [10] (Table 1). This review summarizes the recent progress on the role of Rho kinase/ROCK and its therapeutic potential in vitreoretinal diseases (Figure 1).

## 2. Clinical Application of a ROCK Inhibitor

Pre-clinical research has indicated that ROCK is an important molecule in the pathogenesis of cardiovascular diseases [11]. Based on these accumulated data, a selective ROCK inhibitor, fasudil, has been used in the clinical setting for cerebral vasospasm and ischemic stroke in Japan and China [12]. In ophthalmology, Honjo et al. have demonstrated lowering of intraocular pressure by ROCK inhibition in rabbits. This is the first report to show a therapeutic potential of ROCK inhibitor in eye disease [13]. In another study, a novel, potent, and selective ROCK inhibitor, ripasudil hydrochloride hydrate (K-115), could undergo a structural change and enhance the stearic affinity of the enzyme for ROCK [14]. The enzyme inhibitory effect of ripasudil is about five to ten times higher than that of the previous ROCK inhibitors such as fasudil and ripasudil which were clinically approved in 2014 as an eye drop for glaucoma in Japan [15].

## 3. Unmet Needs in Vitreoretinal Diseases

Vitreoretinal diseases are a common cause of blindness among working age adults [16, 17]. Anti-VEGF administration is currently the most commonly used treatment option for wet age-related macular degeneration (AMD), macular edema secondary to retinal vein occlusion (RVO), and diabetic macular edema (DME) [18–20]. However, in addition to a need for repeated administration and the possibility of local or systemic adverse complications [21], its wide use is implicated in conditions beyond VEGF inhibition such as fibrosis in AMD, retinal ischemia, and fibrovascular membrane contraction in DR [22–25] (Table 1). Chronic anti-VEGF therapy may also increase medical expenses [26]. Therefore, novel therapies aside from VEGF are needed in the treatment of vitreoretinal diseases [27].

## 4. ROCK as a Therapeutic Target for Diabetic Retinopathy

### 4.1. Role of ROCK in Microvascular Complications in DR

While visual acuity is not always affected in nonproliferative stages of diabetic retinopathy (DR) without DME, DR progression can cause neovascularization, vitreous hemorrhages, preretinal fibrovascular proliferation, and tractional retinal detachment, which can lead to severe vision loss [28]. DR pathogenesis is accompanied by microvascular complications such as hyperpermeability, angiogenesis, microthrombosis, and inflammation [29, 30]. Diabetic retinal capillary disorder may be associated with retinal leukocyte stasis (leukostasis) at early nonproliferative stages of DR [31–34]. Leukostasis is mediated by adhesion molecules, intercellular adhesion molecule-1 (ICAM-1), and leukocyte *β*2 integrins (CD18/CD11a and CD18/CD11b) [31, 35]. ROCK pathway has been reported to regulate the expression and function of ICAM-1 in endothelial cells [36] and could be activated in vascular cells by serum from diabetic retinopathy patients [37]. This observation suggested that endothelial cells in diabetic retinopathy patients could be in a “ROCK-activated status” at the systemic level. Furthermore, a study with streptozotocin-induced diabetic model confirmed activation of the Rho/ROCK pathway in retinal microvessels [38]. Moreover, intravitreal fasudil significantly reduced ICAM-1 expression, leukocyte adhesion, and the number of damaged endothelial cells in retinas of diabetic rats [38] (Table 2). These data indicate that ROCK signaling plays important roles in the pathogenesis of microvascular complications in diabetic retinopathy, and its inhibition may represent a new strategy for managing early stage diabetic retinopathy, which is an observation period with no ophthalmic treatment.

### 4.2. Controversial Role of ROCK in Hyperpermeability and Angiogenesis

VEGF plays a critical role in the pathogenesis of DR-related hyperpermeability and angiogenesis [39]. While ROCK inhibition by Y27632 could block VEGF-induced endothelial hyperpermeability [40], the role of ROCK in TNF-*α*-induced endothelial permeability is still controversial [41, 42]. The effect of ROCK inhibitors on hyperpermeability in diabetic retinopathy may be different for each case. A ROCK inhibitor, Y27632, blocked VEGF-induced angiogenesis in an oxygen-induced retinopathy (OIR) model [43], while fasudil inhibited angiogenesis in corneal and OIR models [44, 45] (Table 2). In vitro, ROCK inhibition by fasudil significantly inhibited VEGF-induced retinal endothelial cell proliferation and migration in human and bovine retinal endothelial cells [44, 45]. These previous data suggest that a mechanism of ROCK inhibition on VEGF-induced angiogenesis could be via blockade of endothelial migration and proliferation. In contrast, a study with a ROCK inhibitor H-1152 showed increased VEGF-induced angiogenesis in an OIR model and an in vitro sprouting model via ERK1/2 activation [46]. This discrepancy might be due to different drug affinities against the two ROCK isoforms or an unexpected nonspecific effect [47]. It has also been reported that ROCK signaling could upregulate VEGF in diabetic retina [48].

### 4.3. ROCK as a Therapeutic Target in Proliferative Membrane

In the later stages of DR, epiretinal fibrovascular membranes that form along with retinal neovascularization contract and result in traction retinal detachment (TRD) [49]. ROCK inhibition effectively disrupted *α*-SMA organization and blocked contraction of the proliferative membrane in an in vivo experimental rabbit model [50] (Table 2). In hyalocyte-containing collagen gel assays, ROCK inhibition almost completely abolished PDR vitreous-induced collagen gel contraction mediated through MLC phosphorylation suppression [50, 51].

### 4.4. Involvement of ROCK Different from VEGF in Diabetic Macular Edema

Diabetes reduces occludin quantity at tight junctions in retinal endothelial cells and causes tight junction protein disorganization in retinal arterioles and capillaries [52], presumably leading to vascular hyperpermeability and DME. The Rho/ROCK pathway has been associated with tight junction protein degradation and blood-brain barrier disruption [53]. Furthermore, recent clinical observations suggested that combination therapy of bevacizumab and fasudil intravitreal injection was effective based on structural and functional outcomes in eyes with severe DME that were resistant to current anti-VEGF therapy [54, 55], indicating that ROCK inhibition is mechanistically different from anti-VEGF therapy.

### 4.5. ROCK Inhibition for Retinal Ischemia

Currently, there is no effective treatment for microthrombosis and retinal ischemia. Although laser photocoagulation has been used to treat diabetic retinopathy patients with ischemic retinal tissue, this treatment could cause several adverse events including night blindness. A recent paper showed ROCK inhibition by ripasudil could cause intraretinal vascularization while inhibiting preretinal angiogenesis, leading to reduced hypoxic area in an OIR model [45]. Furthermore, the ripasudil treatment could improve retinal vascular perfusion and induce pericyte coverage [45] (Table 2). This phenomenon could be the vascular normalization that has been proposed in cancer research [56]. However, further investigation using other ROCK inhibitors would be necessary to validate the induction of vascular normalization. Fasudil has already been shown to improve ischemia in patients with acute ischemic stroke [57]. It has been previously reported that ROCK inhibition could cause retinal vessel dilation, and this in turn could contribute to ischemia improvement [58]. A recent study in cats showed that intravitreal ripasudil injection could significantly increase retinal blood velocity and flow [59]. ROCK inhibition may therefore be a new therapeutic strategy for retinal ischemia in retinal vascular disorders.

## 5. ROCK as a Therapeutic Target for Age-Related Macular Degeneration

### 5.1. ROCK2-Mediated Macrophage Polarization in Aging

There are two types of AMD, a dry form that ultimately leads to macular atrophy and a wet and exudative form characterized by choroidal neovascularization (CNV) and leakage [60]. The pathogenesis of AMD remains incompletely understood. Macrophages are found in CNV lesions and have been reported to promote and inhibit CNV [61, 62]. This phenotype-associated mechanism was unknown. Furthermore, it was also unclear how aging promotes the pathogenesis. A recent paper by Zandi et al. showed that macrophage polarization was triggered by ROCK2 signaling, which is increased with age, and a shift of the fundus microenvironment through selective ROCK2 inhibition improved the pathology [63] (Table 2).

### 5.2. ROCK as a Possible Target in Subretinal Fibrosis

Wet AMD-related CNV eventually causes fibrosis that could lead to irreversible vision loss [64, 65], and there is currently no effective treatment for this fibrosis. A ROCK inhibitor, AMA0428, was recently reported to be effective in reducing fibrosis in a mouse CNV model [66] (Table 2). As the Rho/ROCK pathway is a downstream signaling of fibrotic disease drivers, such as TGF-*β* [67, 68], ROCK inhibition might block TGF-*β*-related subretinal fibrosis although the detailed mechanism is still unknown. ROCK inhibition may therefore be a new therapy for fibrosis and neovascularization in AMD.

## 6. ROCK as a Therapeutic Target for Proliferative Vitreoretinopathy

Proliferative vitreoretinopathy (PVR) is the leading cause of failure after retinal detachment surgery. PVR is characterized by the growth and contraction of cellular membranes within the hyaloid and retina and on both retinal surfaces following retinal reattachment surgery [69]. Retinal detachment allows macrophages, retinal pigment epithelial (RPE) cells, glial cells, and fibroblasts to migrate to the vitreous, where they proliferate, survive, form extracellular matrix proteins, and assemble into a membrane [70]. Some studies suggest that cytokines such as TGF-*β*2 and PDGF contribute to PVR pathogenesis [71]. However, there is currently no effective treatment other than surgery. Various recent papers have shown that the ROCK pathway is involved in PVR pathogenesis. The importance of ROCK for TGF-*β*-induced gel contraction by retinal pigment epithelium has been reported [67, 72–74]. Furthermore, in vivo studies suggest that ROCK inhibition could block TRD development [50, 73] and that ROCK inhibitors might aid in PVR prevention and development apart from vitrectomy surgery [75] (Table 2).

## 7. Future Directions of ROCK Inhibitors: Neuroprotection

Microvascular changes underlie DR and AMD, while histological studies have characterized the loss of neurons [76]. The roles of neural retinal alterations in the pathogenesis of early retinopathy and the mechanisms of vision loss have been emphasized [77]. A recent report has demonstrated that administration of an oral ROCK inhibitor, K115, delayed RGC death [78]. Fasudil also resulted in ischemia-related apoptosis of retinal cells by inhibiting Bax/Bcl-2, caspase-3, and iNOS in rats [79]. However, the importance of ROCK for neural degeneration in vitreoretinal diseases including DR and AMD is unknown. Future investigations are expected to demonstrate a therapeutic potential of ROCK inhibitors in vitreoretinal disorders.

## 8. Future Directions of ROCK Inhibitors: Beyond VEGF

In summary, some disease states extend beyond VEGF inhibition, including fibrosis in AMD, retinal ischemia, retinal neuropathy, and fibrovascular membrane contraction in DR (Table 1). ROCK inhibition may be effective in these pathological conditions. A previous study using radio-labeled drug revealed that ripasudil could reach the retina and choroid after eye drop administration in rabbits [14]. If proven effective, topical ophthalmic treatment would be beneficial for patients with vitreoretinal diseases. Furthermore, the role of ROCK isoforms in vitreoretinal diseases is unclear. In a recent paper, a ROCK2 inhibitor, but not the pan-ROCK inhibitor fasudil, was beneficial in age-related immune changes in AMD [63]. Intensive investigation is needed to elucidate the role of ROCK isoforms in the pathogenesis of these vitreoretinal diseases.

## 9. Conclusion

The clinical application of anti-VEGF therapy and its success constitutes the beginning of the era of molecular targeting drugs in ophthalmology. Currently, various molecular targeting drugs are under clinical trials for vitreoretinal diseases [80]. Several will be clinically applied in the near future, and these are expected to impact the therapeutic strategy of vitreoretinal diseases. ROCK could be one of these potential drug targets. An optimal administration method/administration protocol is expected to emerge based on both clinical and nonclinical investigations.

## Figures and Tables

**Table 1:** Comparison between VEGF and Rho/ROCK in disease pathogenesis.

Biological process	VEGF inhibition	Rho/ROCK inhibition
Ischemia	Possible induction of ischemia [24]	Vascular normalization via pericyte coverage [45]; vessel dilation [58]; increased blood velocity and retinal blood flow [59]
Angiogenesis	Antiangiogenesis [81, 82]	Antiendothelial proliferation in vitro [44, 45]; antiendothelial migration in vitro [44, 45]; antiangiogenesis in vivo retina [43, 45]; antiangiogenesis in vivo choroid [63, 66]
Hyperpermeability	Antipermeability [83, 84]	Antipermeability in choroidal neovascularization [63, 66]
Inflammation	Antileukocyte trafficking [81]; antileukostasis [84]	Antileukostasis [38]; anti-M2 macrophage [63]
Membrane contraction	Possible induction of membrane contraction and tractional retinal detachment [23]; vessel contraction [25]	Inhibition of membrane contraction in vivo [50, 73]; reduced collagen synthesis in RPE [66]; inhibition of gel contraction by RPE [72, 73]; anti-RPE proliferation [72]; actin depolymerization in RPE [74]
Neuronal damage	Possible induction of photoreceptor damage [85, 86]	Neuroprotection of RGC [78, 87, 88]
Fibrosis	Risk of inducible fibrosis [22, 65]	Antifibrosis in choroidal neovascularization [66]

**Table 2:** ROCK inhibitors in animal models of vitreoretinal diseases.

Animal model	OIR model (oxygen-induced retinopathy)	STZ model (streptozotocin-induced diabetes model)	CNV model (choroidal neovascularization model)	PVR (proliferative vitreoretinopathy model)
Fasudil	Antiangiogenesis [45]	Antileukostasis [38]	Antipermeability [63]; anti-M2 macrophage [63]; antiangiogenesis [63]	Inhibition of membrane contraction [50]
Ripasudil (K115)	Vascular normalization via pericyte coverage [45]; antiangiogenesis [45]	No report	No report	No report
Y27632	Antiangiogenesis [43]	No report	No report	Inhibition of membrane contraction [73]
AMA0428	Antiangiogenesis [89]; inhibition neuronal cell death [89]	Antileukostasis [89]; antipermeability [89]; neuroprotection of RGC [89]	Antiangiogenesis [66]; antifibrosis [66]; antiinflamation [66]	No report
